# Trichogerminoma, a rare cutaneous follicular neoplasm with indolent clinical course: report of two cases and review of literature

**DOI:** 10.1186/1746-1596-8-210

**Published:** 2013-12-19

**Authors:** Li-li Chen, Jin-tao Hu, Yang Li

**Affiliations:** 1 Department of Pathology, The First Affiliated Hospital, Sun Yat-sen University, 58, Zhongshan Road II, Guangzhou 510080, China; 2 Department of Pathology, Shenzhen People’s Hospital, 1017, Dongmen Road, North Shenzhen 518020, China

**Keywords:** Trichogerminoma, Trichoblastoma, Hair follicular neoplasm

## Abstract

Trichogerminoma is a rare cutaneous adnexal neoplasm of the hair germ cell and usually associated with benign clinical course and favorable outcome. Since its first description by Sau et al. in 1992, only a few cases have been reported up to date. Herein, we report two additional cases occurring in the hip and right thigh, respectively. Both patients are male, one is 78 years old, the other is 29 years old. Histological examination reveals well-circumscribed dermal nodule composed of lobules of basaloid cells with surrounding pseudocapsule. The distinct characteristic of the tumor is that most of the lobules display a special pattern of round nests or cell balls arranged in the central part with the peripheral palisading. Immunostaining showed ring-like fashion of CK5/6, P63 and Bcl-2 with negative or weak staining in the “cell balls”. There was no recurrence after complete excision during the period of follow-up. To the best of our knowledge, this is the first report of trichogerminoma in Chinese population. In contrast to the previously reported cases, ours present the similar morphological features with distinct immunohistochemical characteristics. We consider the concept of trichogerminoma exists with no doubt by its identifiable morphological features, and it should be classified as a variant of trichoblastoma. Because of its malignant potential, complete excision is a prior choice of treatment for this rare but distinctive tumor.

**Virtual slides:**

The virtual slide(s) for this article can be found here: http://www.diagnosticpathology.diagnomx.eu/vs/1558612241110439.

## Background

Trichogerminoma is a rare cutaneous follicular tumor with differentiation towards the hair germ epithelium. In 1992, Sau et al. reported 14 cases of a benign hair germ neoplasm and firstly proposed a brandly new term of trichogerminoma [1]. Only four additional cases have been reported since the first description [2-5]. Most reports of trichogerminoma have come from America, Europe and Korea with a slight male bias. Histologically, trichogerminoma is characterized by well-demarcated nodules composed of basaloid cells with concentrically arranged round nests or cell balls in the central parts and peripheral palisading. It is difficult to distinguish this tumor from other hair-originated tumors, such as trichoblastoma, trichoepithelioma, basal cell carcinoma and tricholemmoma. Because of similarity in histological appearance and overlapping in immunohistological profiles, it is a great challenge for pathologists to make a definite diagnosis accurately. Clinically, trichogerminoma could be confused with epidermal cyst, trichoepithelioma, and basal cell carcinoma. We present herein two cases of trichogerminoma with benign clinical behavior. The histological and immunohistochemical features of this tumor, as well as differential diagnosis are discussed.

## Case presentation

### Patients and clinical management

#### Case 1

A 78-year-old Chinese male patient presented with a 10-year history of a subcutaneous solitary nodule on the left hip without clinical symptoms. The skin above the nodule had no difference with other areas, and it was a hemispheric, palpable, well-demarcated, movable nodule. The pre-operative diagnosis was suspected sebaceous cyst. The nodule was totally resected by surgery.

#### Case 2

A 29-year-old Chinese male patient was referred for orthopedic surgery for removal of a mass on right thigh with 3-year history, which had recently grown in size. Examination revealed a single boundary clear protuberant, which was non-ulcerated, firm, movable and 2 cm in diameter without any clinical symptoms. The pre-operative diagnosis was epidermal cyst/dermatofibroma. The nodule was totally resected.

## Material and methods

The surgical specimens of patients were routinely fixed in 10% neutral buffered formalin. The tissues were embedded in paraffin. Four micrometer-thick sections were stained with hematoxylin and eosin. Immunohistochemical analyses were performed using the ChemMate Envision/HRP Kit (Dako, Glostrup, Denmark). The antibodies used in this study were pan-CK (AE1/AE3), CK5/6, CK7, CK20, Bcl-2, CD10, P63, CD34, S-100, calretinin and Ki-67. The antibodies were obtained from Dako Cytomation (Carpintaria, CA) and Santa Cruz Biotechnology (Santa Cruz, CA). Slides were dewaxed and rehydrated routinely and then were treated with 10 mmol citrate buffer (pH 6.0) in a microwave for antigen retrieval. After incubation with diluted primary antibodies, slides were treated with the ChemMate Envision/HRP Kit for 30 minutes at room temperature followed by development with diaminobenzidine (DAB) for visualization.

### Histological findings

#### Case 1

Under microscopic examination, the surgical specimen showed a sharply circumscribed, symmetric nodule composed of multiple lobules with a fibrous pseudocapsule. The neoplasm was located in the deep dermis with no connection to the superficial epidermis. The lobules were made up of basophilic epithelial cells separated by a fibrocytic myxoid stroma. There were no clefts separating the tumor cells and the surrounding stroma, but stroma-stroma clefts could frequently be seen. Most of the lobules had the distinctive appearance of round nests or cell balls arranged in the central part. These nests formed by pale cells with disperse chromatin and relatively aboundant cytoplasm, which occupied most areas of the lobules, with only a peripheral rim of palisading basaloid cells. Mitotic figures and apoptotic cells were observed occasionally. In a few fields, well-differentiated keratinizing folliculocystic structures were present. Irregular cords of basaloid cells extended from the periphery of the lobules and form buds into stroma. The myxoid stroma showed a moderate number of fibroblasts and mast cells, and inflammatory infiltration of some lymphocytes and mononuclear cells was observed surrounding the epithelial lobules.

**Figure 1:**
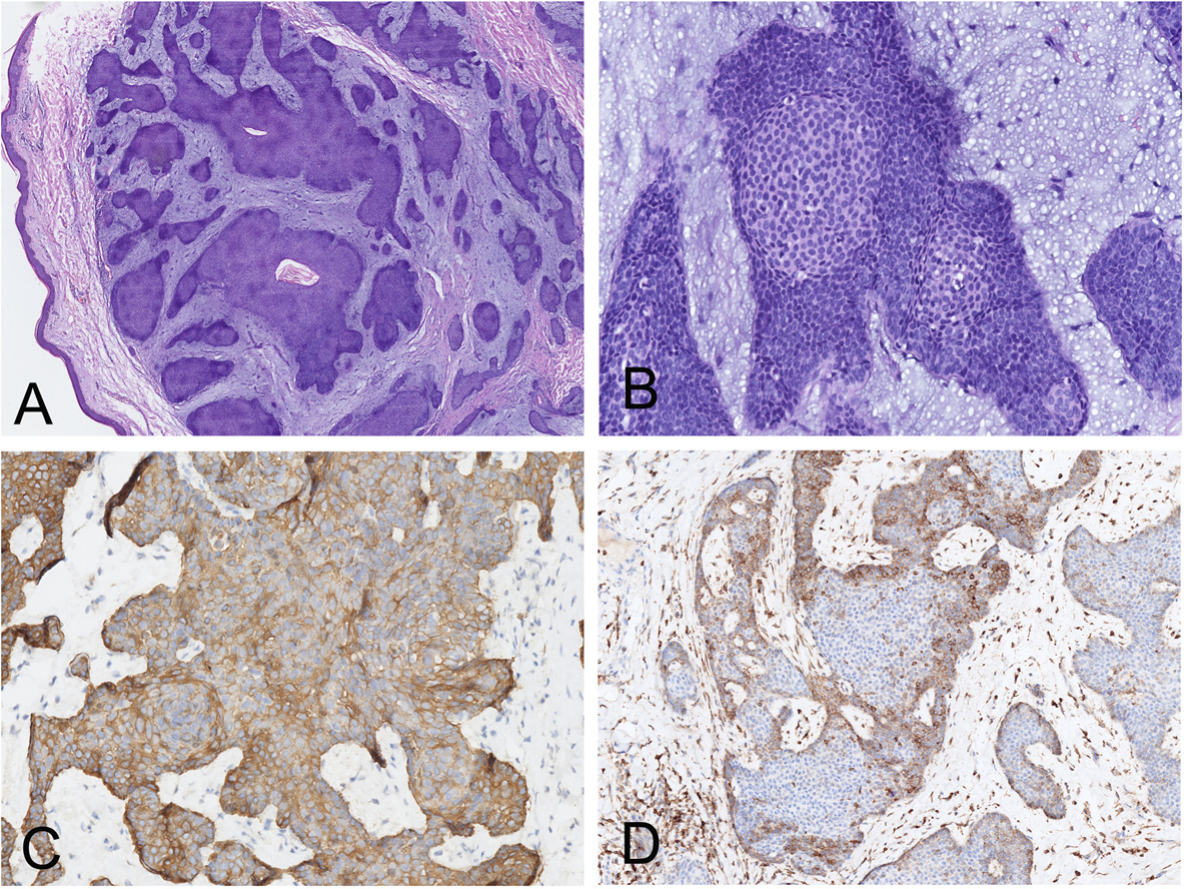
**Photomicrograph of the skin lesion of case 1. (A)** Low-power view showed a well-circumscribed epidermal mass composed of basaloid lobules with a fibrous pseudocapsule. **(B)** Distinct appearance of centrolobular cell balls with undifferentiated basaloid cells displaying peripheral palisading and myxiod stroma. **(C)** CK5/6 immunoreactivity was more intense in the outer layer tumor cells than those within the round nests. **(D)** The peripheral rim of palisading basaloid cells were stained with Bcl-2, along with the central part non-stained, giving the appearance of rings.

Immunohistochemical staining showed the tumor cells were positive for pan-CK (AE1/AE3), CK5/6, P63, and focally positive for Bcl-2, CD10, epithelial membrane antigen (EMA) whereas they were negative for CK7, CK20, carcinoembryonic antigen (CEA) and calretinin. The myxoid stroma around the nests displayed CD34 and CD10. A conspicuous phenomenon was that the peripheral rim of palisading basaloid cells were stained with Bcl-2, CD10, along with the central part nonstained. CK5/6 had a slightly weaker staining in the round nests or cell balls than the outer layer of the lobules (Figure 1). Small aggregates of the tumor cells expressed calretinin-positive in some lobules. Merkel cells scattered within the lobules and the epidermis around the tumor were revealed by staining for CK20 antibody. Ki67 highlighted a low nuclear proliferative rate (less than 10%).

#### Case 2

Histological examination revealed a well-demarcated, deep dermal and subcutaneous lesion made up of multi-lobular basaloid proliferation and a fibrotic stroma. The tumor was separated from the surrounding soft tissue by a thin capsule and was not connected with the overlying epidermis. Tumor-stroma retraction artifacts were not observed. The neoplasm was composed of basophilic epithelial cells displayed palisading. Scattered folliculocystic structures and areas of calcification were identified as calcified folliculocystic structures. Focal pigmented material was also present. Characteristically, some of the loblules had concentrically, densely packed round nests or cell balls with vesicular nuclear, prominent nucleoli and disperse chromatin, and the outer layer of the lobules was comprised of undifferentiated columnar basaloid cells displaying peripheral palisading appearance. In some areas, strands and cords of germ cells extended from the periphery of the lobules. High magnification of a papillary mesenchymal body showed invagination of cellular fibroblastic stroma into peripheral bulb-like area of the tumor.

**Figure 2:**
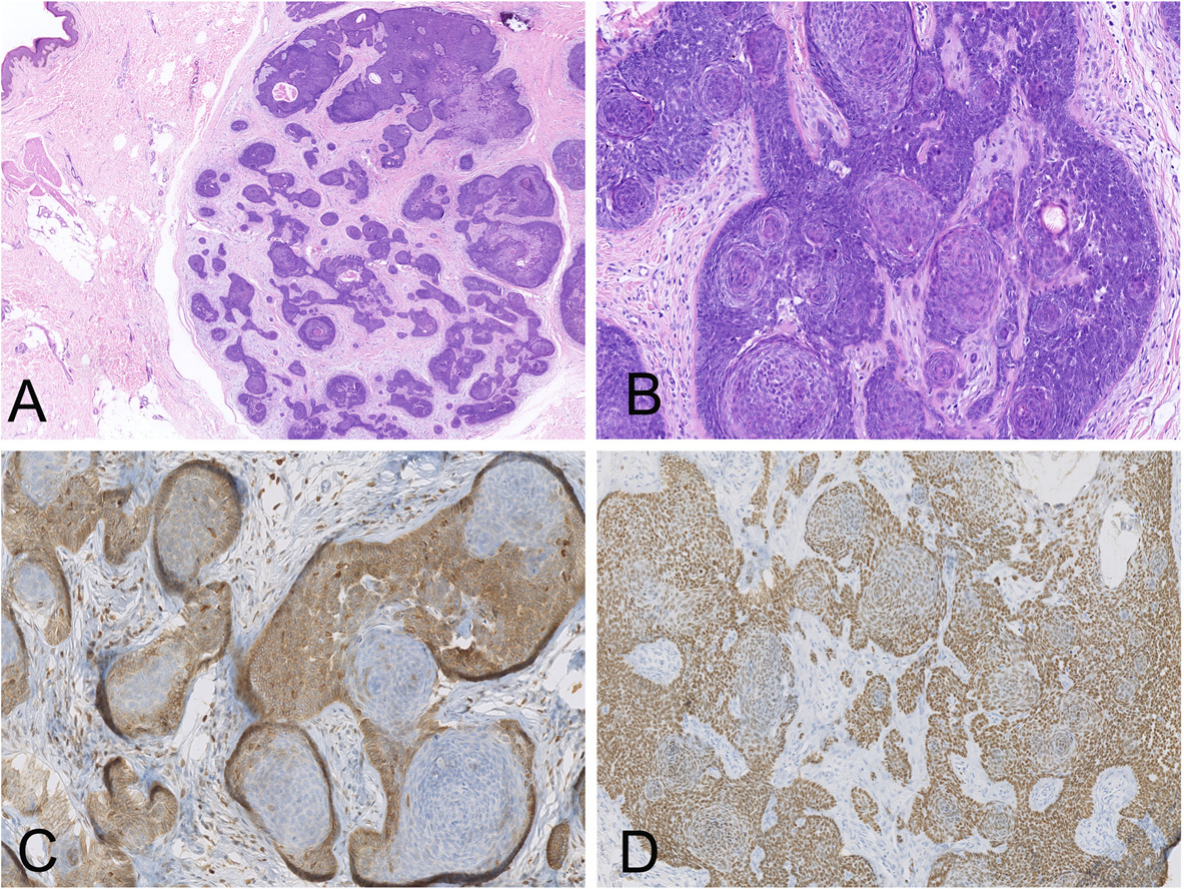
**Photomicrograph of skin lesion of case 2. (A)** Low magnification showed a sharply demarcated tumor made up of multiple basaloid lobules in the deep dermis. **(B)** Concentrically, densely packed round nests or cell balls of pale cells in the central part of the lobules with peripheral palisading. **(C)** Bcl-2 was stained prominently in the out layer tumor cells with the centrolobular cell balls non-stained. **(D)** P63 showed weaker staining in the central part than those in the periphery.

Immunohistochemically, the tumor cells were stained with antibodies to pan-CK (AE1/AE3), and P63, focally stained with antibody to Bcl-2 and CD10, but not with CK7. As a remarkable feature with the majority of the lobules, the round nest or cell balls were not stained while the outer layer displayed prominently positive to the anti-Bcl-2. The expression of CD10 was similar to that of Bcl-2, but just in a few areas. P63 had a relatively weaker staining in the central part than in the periphery (Figure 2). There were Merkel cells and dendritic cells scattered within the lobules and the epidermis around the tumor, which disclosed separately by staining for anti-CK20 and anti-S-100 proteins. The Ki67 index is lower (less than 5%).

Based on the histopathological findings, both two cases were consistent with the diagnostic criteria of trichogerminoma according to Sau’s description [1].

The post-operative recovery was uneventful. The patients were discharged from hospital and on a regular follow-up for 6 and 12 months, respectively. No recurrence of the tumors was observed within the periods of follow-up.

## Discussion

Trichogerminoma is a rare cutaneous adnexal neoplasm that simulates hair germ cell structures. In 1992, Sau et al. reported 14 cases of a benign hair germ neoplasm under the new term of trichogerminoma [1]. All the cases in their study displayed a unique constellation of histological characteristics which did not precisely match any of the previously described tumors of hair follicular tumors. Thus the authors considered the neoplasm to be a distinct entity and designated it as “trichogerminoma”. Before 1992, tumors similar to trichogerminoma may have been reported as trichoblastic fibroma by some authors. Grouls and Hey reported 6 cases of trichoblastic fibromas which microscopic features are identical to those seen in trichogerminoma [6]. Since the term of trichogerminoma was initially proposed, Kazakov et al. described an additional case confirming the uniqueness of the neoplasm and pointed subtle immunohistochemical differences between trichogerminoma and trichoblastoma in 2002 [2]. Subsequently, there were only 3 cases of trichogerminoma reported separately in the next decade and Tellechea & Reis discussed its position within the spectrum of the hair germ neoplasms in their report [3-5]. In 2002, the term of trichogerminoma was approved by WHO classification of skin tumors as a synonym for trichoblastoma [7].

By reviewing all the English literature we can retrieve, only 20 cases have been reported, including our 2 cases (Table 1) [1-5]. Taken together all the reports, trichogerminoma mainly affects elderly people (mean age: 52 years) and it shows a slight male bias, with the male: female ratio approximately being 2:1. The main clinical presentation was a single solitary, slowly growing, dermal or subcutaneous nodule without any clinical symptoms. The tumors located on the head and neck, trunk, extremities and hip. Most of reported cases exhibited a benign biological behavior and had no recurrence or metastasis after complete surgical excision in varying follow-up periods, although transformation into high-grade carcinoma with metastatic disease and death has also been reported [1]. In our present cases, histological features were identical to those described in trichogerminoma, which showed the lobules composed of the basaloid epithelial cells, characteristic cell balls, and hair follicular differentiation of varying degrees. The immunophenotype of our cases shows that the ring-like pattern of the expression of CD10 and Bcl-2 in some parts with prominent staining in the peripheral parts and negative in the concentric rounded nests, and that the relatively weaker staining of CK5/6 and P63 in the central parts than in the periphery. These immunostaining characteristics show the differences between our cases with the previous reported ones.

**Table 1:** Clinicopathological characteristics of trichogerminoma described in present and previous reports

**Case no.**	**Author (year)**	**Diagnosis**	**Age (year)/ gender**	**Site**	**Immunoprofiles**	**Treatment**	**Outcome**
1	Sau G (1992) [1]	TG	34/female	Scalp	NA	TSR	NED 6 years
2		TG	55/female	Back	NA	TSR	NED 11 years
3		TG	53/male	Right calf	NA	TSR	NED 9 years
4		TG	16/female	Right eye brow	NA	TSR	NA
5		TG	25/male	NA	NA	TSR	NED 8 years
6		TG	27/female	Forehead	NA	TSR	NED 7 years
7		TG	60/male	Right cheek	NA	TSR	NED 5.5 years
8		TG	53/male	Submental	NA	TSR	NED 6 years
9		TG	59/female	Left ear	NA	TSR	NED 6 years
10		TG	43/male	Abdomen	NA	TSR	NED 3 years
11		TG	73/male	Right breast	NA	TSR	NED 3 years
12		TG	73/male	Abdomen	NA	TSR/RT	Recur & metast to soft tissue LN & liver 4 months
13		TG	58/male	Right hip	NA	TSR	NED 2 years
14		TG	61/male	Left arm	NA	TSR	NED 1.5 years
15	Kazakov (2002) [2]	TG	41/male	Left eye	AE1/AE3 +, CK20 -	TSR	NED 6 months
16	Pozo (2005) [3]	TG	74/female	Face	AE1/AE3 +, CK20 -	TSR	NA
17	Tellchea (2009) [4]	TG	45/male	Scalp	AE1/AE3 +, CK5/6 zonal+	TSR	NED 10 years
18	Kim (2010) [5]	TG	79/female	Neck	AE1/AE3 +, CK5/6 zonal+	TSR	NED 6 months
19	Present case No.1	TG	78/male	Hip	AE1/AE3 +, CK5/6, Bcl-2 zonal+, CK20 -	TSR	NED 6 months
20	Present case No.2	TG	29/male	Right thigh	AE1/AE3 +, CK5/6, P63, Bcl-2 zonal+, CK20 -	TSR	NED 12 months

TG, trichogerminoma; NA, not available; NED, no evidence of disease; TSR, totally surgical resection; RT, radiotherapy.

Histologically, trichogerminoma is a well-circumscribed dermal-subcutaneous benign follicular neoplasm. It is characterized by multiple lobules composed of basaloid cells with concentrically arranged rounded nests or cell balls in the central parts and peripheral condensation. Inmmunohistochemically, the characteristic features of trichogerminoma are that the ring-like pattern of the CK5/6 expression with prominent staining in the peripheral parts and negative in the concentric rounded nests, and that the negative staining of CK20. In the present cases, the expression of CK5/6 in case 1 showed a certain pattern of ring with prominent immunoreactivity in the peripheral rims of the lobules, whereas the contrast between the central and the peripheral cells of the epithelial nests which in our cases were not so intense as that reported previously. P63 in case 2 had a relatively weaker staining in the central part than in the periphery when no regional difference was found in the staining pattern between the two types of the tumor cells in case 1 and the previously reported cases [5]. The tumors of both cases were negative for CK20 and S-100. Interestingly, expression of CD10 and Bcl-2 were expressed at the outermost epithelial cell layer of the tumor lobules in a few areas. However, the lesions of two cases exhibited the typical morphologic appearance of trichogerminoma which was consistent with diagnostic criteria of trichogerminoma despite of the small differences in the immunohistochemical expression of CK5/6 and P63.

Trichogerminoma should be distinguished from other trichogenic tumors made up of basoloid cells or hair follicular differentiation. The mainly differential diagnosis includes trichoblastoma, trichoepithelioma, basal cell carcinoma and tricholemmoma. Trichoblastoma is generally considered to be composed of basaloid cells and to display less differentiation towards hair follicular structures than trichogerminoma. The characteristic features of trichogerminoma, with central lobular areas of primitive pale cells, have been observed in trichoblastoma [8], but this pattern is not the dominant microscopic appearance. Trichoepithelioma is histologically characterized by islands of basaloid cells and infundibulocystic keratinized structures. Trichoepithelioma differs from trichogerminoma by its lack of concentric rounded nests in the lobules. Basal cell carcinoma is made up of proliferative basaloid cells with peripheral palisading, which usually shows tumor-stroma retraction spaces and attachments to the overlying epidermis. Although follicular differentiation sometimes can be observed in basal cell carcinoma, its absence of papilla formation and centrolobular pale cells is the main identification. Tricholemmoma is a benign clear cell adnexal neoplasm with outer root sheath differentiation. Microscopically, the neoplasm is composed of glycogen-rich clear cells with the columnar cells at the periphery that rest upon a thickened basement membrane (Table 2).

**Table 2:** Differential diagnosis for trichogerminoma in histological and immunohistochemical characteristics

**Characteristics**	**Trichogerminoma**	**Trichoblastoma**	**Trichoepithelioma**	**Basal cell carcinoma with follicular differentiation**	**Trichilemmoma**
Basic histological features	Circumscribed lobules made up of basaloid cells with peripheral palisading, no epidermal connection, no retraction space	Large basaloid epithelial lobules, no epidermal connection, no retraction cleft	Mutiple lobules and nests of basaloid cells, confined to superficila dermis, no retraction cleft	Irregular lobues made up of basaloid cells with prominent peripheral palisading, with epidermis attachment and retracion space	Irregular lobues, with epidermis and follicles attachment
Specific characteristics	Centrolobular cell balls, papillary mesenchymal bodies(++), follicular cyst(+/−),	Papillary mesenchymal bodies(++), follicular cyst(+/−)	Papillary mesenchymal bodies(+), horn cyst(++)	Mitotic and apoptotic bodies(+), follicular cyst(+/−), papillary mesenchymal bodies(−)	Thickened, eosinophilic basement membrane surrounding tumor lobules
Immunohistochemistry	“Zonal” CK5/6, P63 and Bcl-2 immunostaining, CK20+ Merker cells in lobules	“Zonal” Bcl-2 immunostaining, CK20+ Merker cells in lobules	Androgen receptor -, CK20+ (in some reports)	Bcl-2 diffusely(+), No CK20+ Merker cells in tumor lobules	PAS + clear cells

It is still controversial whether trichogerminoma should be classified apart from other hair germ tumors. Most dermatopathologists considered it should be within the spectrum of trichoblastoma. However, individual histological features of round, densely packed pale centrolobular cells distinguish trichogerminoma from trichoblastoma and deserve to its recognition. The immunostaining fashion of CK5/6, CK14 and P63 which were not present in trichoblastoma also support this point of view. Admittedly, there are some similarities between trichogerminoma and trichoblastoma, including the profile of Bcl-2 and CD10 expression [9-11]. Reviewing all the cases previously reported and our two cases, we come to a conclusion that maybe trichogerminoma should be an independent concept, as a histological subtype of trichoblastoma, since they have basic common features and distinct differences.

Trichogerminomas generally behave in a benign fashion. There was no recurrence after surgical removal with only one exception that followed by malignant transformation to undifferentiated carcinoma and died of metastasis [1]. So, complete excision is recommended because of the malignant potential. Combined with the follow-up of our cases, trichogerminomas have a good prognosis.

## Conclusion

Herein we report two extremely rare cases of trichogerminoma. To our best knowledge, the presenting cases are the first report in Chinese population. In contrast to the previously reported cases, ours present the similar morphological features with distinct immunohistochemical characteristics. According to the report, we consider that trichogerminoma should be an independent concept, as a histological variant of trichoblastoma, since they have basic common features and distinct differences. Because of its rarity, the careful inspection under the microscopy and adequate immunohistochemical examination are recommended for helping to make an accurate diagnosis.

### Consent

Written informed consent was obtained from the patients for publication of this case report and any accompanying images. A copy of the written consent is available for review by the Editor-in-Chief of this journal.

## Competing interests

The authors declare that they have no competing interests.

## Authors’ contributions

LC and JH made contributions to acquisition of clinical data, and analysis of the histological features by H&E staining. They are joint first co-authors and made an equal contribution to this work. YL revised manuscript critically for important intellectual content and had given final approval of the version to be published. All authors read and approved the final manuscript.
